# RNA binding protein SAMD4: current knowledge and future perspectives

**DOI:** 10.1186/s13578-023-00968-x

**Published:** 2023-02-02

**Authors:** Xin-Ya Wang, Li-Na Zhang

**Affiliations:** Beijing International Science and Technology Cooperation Base of Antivirus Drug, Faculty of Environment and Life, Beijing University of Technology, 100124 Beijing, People’s Republic of China

**Keywords:** SAMD4A, SAMD4B, Smaug, RNA-binding protein, SAM domain, Post-transcriptional regulator, Translational repressor

## Abstract

SAMD4 protein family is a class of novel RNA-binding proteins that can mediate post-transcriptional regulation and translation repression in eukaryotes, which are highly conserved from yeast to humans during evolution. In mammalian cells, SAMD4 protein family consists of two members including SAMD4A/Smaug1 and SAMD4B/Smaug2, both of which contain common SAM domain that can specifically bind to different target mRNAs through stem-loop structures, also known as Smaug recognition elements (SREs), and regulate the mRNA stability, degradation and translation. In addition, SAMD4 can form the cytoplasmic mRNA silencing foci and regulate the translation of SRE-containing mRNAs in neurons. SAMD4 also can form the cytosolic membrane-less organelles (MLOs), termed as Smaug1 bodies, and regulate mitochondrial function. Importantly, many studies have identified that SAMD4 family members are involved in various pathological processes including myopathy, bone development, neural development, and cancer occurrence and progression. In this review, we mainly summarize the structural characteristics, biological functions and molecular regulatory mechanisms of SAMD4 protein family members, which will provide a basis for further research and clinical application of SAMD4 protein family.

## Introduction

Sterile alpha motif domain containing protein 4 (SAMD4) is a novel class of post-transcriptional regulators and translational repressors in eukaryotes, which is homologous to Smaug protein in *Drosophila melanogaster* [1, 2]. Smaug is identified to be a sequence-specific RNA-binding protein that is mainly involved in the regulation of post-transcription, mRNA stability and translation repression during early embryonic development of *Drosophila melanogaster* [3–5]. Smaug protein is highly conserved from yeast to humans during evolution, and its homology with yeast Vts1p and mammalian SAMD4 indicates that they have certain similar characteristics in structure and function [6]. In mammalian cells, the identified SAMD4 protein family members include SAMD4A/Smaug1 and SAMD4B/Smaug2. Studies have shown that both SAMD4 family members contain a specific RNA binding domain, the sterile alpha motif (SAM) domain, which leads to directly bind target mRNAs and participate in the post-transcriptional regulation and mRNA translation repression [3, 6]. It is well known that RNA binding proteins are involved in the post-transcriptional regulation processes of gene expression, including mRNA splicing, nuclear export, mRNA translation, mRNA stability regulation and subcellular re-localization [7–9]. In addition, the occurrence of many diseases is caused by a large number of mutations in genes that encode RNA-binding proteins, which is closely associated with these RNA binding proteins mediating the steps in post-transcriptional regulation of gene expression [10]. Current studies have demonstrated that SAMD4 is involved in the regulation of several physiological and pathological processes. For example, SAMD4 can regulate metabolic homeostasis through mechanistic target of rapamycin complex 1 (mTORC1) signaling [11]. SAMD4 also can recruit CCR4 and POP2 deadenylases to target mRNA to trigger their deadenylation and degradation [12]. The epigenetic modification of SAMD4 is related to the transcriptional downregulation of gene expression in human cancer [13]. The abnormal expression of SAMD4 is also correlated to many diseases such as myopathy [11], brain ageing [14], skeleton development [15] and cancer development [13]. The latest studies have revealed that human SAMD4 protein family members suppress human hepatitis B virus (HBV) replication and the expression levels of SAMD4 are associated with HBV sensitivity in humans [16].

Previous studies mainly focused on the investigation of biological functions of the SAM domain of SAMD4 protein and the effects of Smaug on mRNA translation and mRNA stability during the early embryonic development of *Drosophila melanogaster*. With the development of research, many researchers are starting to pay attention to the new physiological and pathological functions of SAMD4 protein family members in mammalian cells and even with human diseases. However, the pathogenic molecular mechanisms between SAMD4 and various diseases still need to be further explored. Therefore, this paper reviews the current research progress on the functional roles and molecular mechanisms of SAMD4 protein family members in recent decades, which will provide possible strategies for further revealing the regulatory mechanisms and therapeutic roles of SAMD4 family members in the related diseases.

## Evolutionary conservation of SAMD4

SAMD4 is a conserved translational repressor from yeast Vts1p to mammalian SAMD4 family [6]. Phylogenetic analysis indicates that SAMD4 family members are evolutionarily conserved, and these proteins have the same motif structure. Among them, human *SAMD4A* and *SAMD4B* genes are located on human chromosome 14 and 19, respectively, which are widely expressed in various human tissues and organs. Sequence analysis results show that the protein sequences of SAMD4A and SAMD4B have a certain homology and conservation in evolution.

### Phylogenetic analysis of SAMD4

**Fig. 1:**
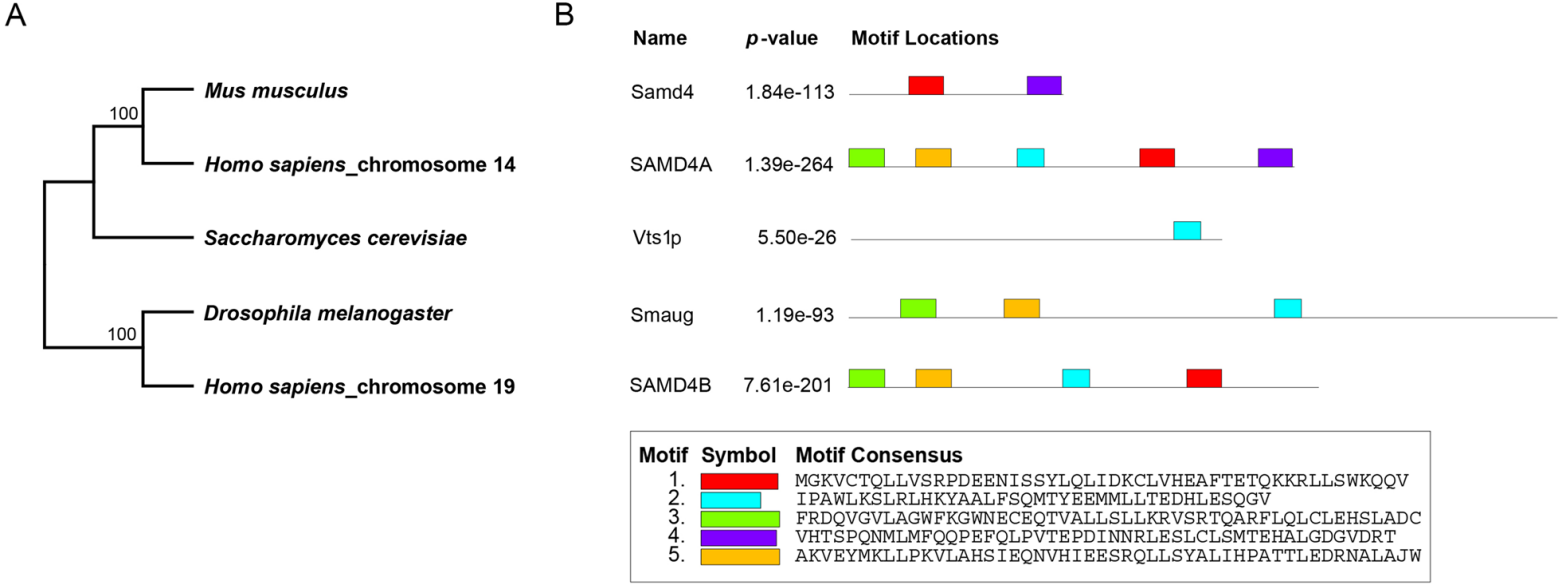
The phylogenetic tree analysis and motif analysis of SAMD4 family members from different species. **A** Phylogenetic tree analysis of SAMD4 in *Saccharomyces cerevisiae*, *Drosophila melanogaster*, *Mus musculus* and *Homo sapiens*. **B** Motif structure analysis of Samd4 (*Mus musculus*), Vts1p (*Saccharomyces cerevisiae*), Smaug (*Drosophila melanogaster*), SAMD4A and SAMD4B (*Homo sapiens*)

According to the sequence information of SAMD4 family members downloaded from National Center for Biotechnology Information (NCBI) database (https://www.ncbi.nlm.nih.gov/), we constructed the phylogenetic tree analysis and the motif analysis from yeast Vts1p to human SAMD4. The phylogenetic tree analysis indicates that SAMD4 is evolutionarily conserved in *Saccharomyces cerevisiae*, *Drosophila melanogaster, Mus musculus* and *Homo sapiens* (Fig. 1 A). Homologous motif analysis from the MEME Suite database (https://meme-suite.org/meme/doc/meme.html) shows that SAMD4 protein family members have the similar motif structure. Among the five protein members, human SAMD4A and SAMD4B have the most common motif structure characteristics, indicating that they have high homology and sequence conservation (Fig. 1B).

### Chromosomal localization and sequence homology of SAMD4

**Fig. 2:**
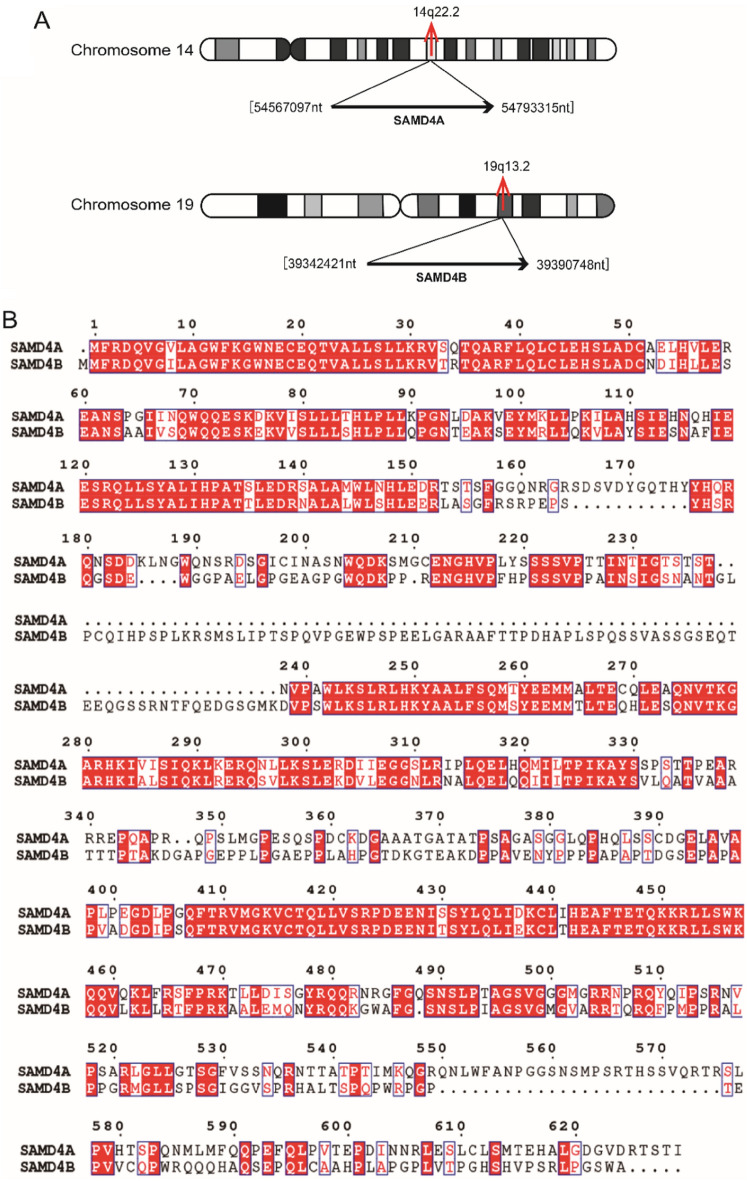
The localization of human SAMD4 family members on human chromosomes and the sequence homology analysis of SAMD4 proteins. **A** SAMD4A is located on chromosome 14q22.2. SAMD4B is located on chromosome 19q13.2. **B** The sequence alignment results of SAMD4A and SAMD4B proteins

According to the query results in human genome from the GEO database (https://www.ncbi.nlm.nih.gov/geo/), *SAMD4A* gene is located on chromosome 14q22.2 with the genome sequence length of about 226, 219 nt, which is highly expressed in testis, heart and brain tissues. Nevertheless, *SAMD4B* gene is located on chromosome 19q13.2 with the genome sequence length of about 48, 328 nt, which is highly expressed in testis, ovary, brain and other tissues (Fig. 2A). The sequence alignment result of Clustal Omega database (https://www.ebi.ac.uk/Tools/msa/clustalo/) shows that the sequence similarity of human SAMD4A and SAMD4B is 41%, suggesting that they are homologous proteins in evolution with high sequence homology (Fig. 2B).

## Domain structure of SAMD4 protein

**Fig. 3:**
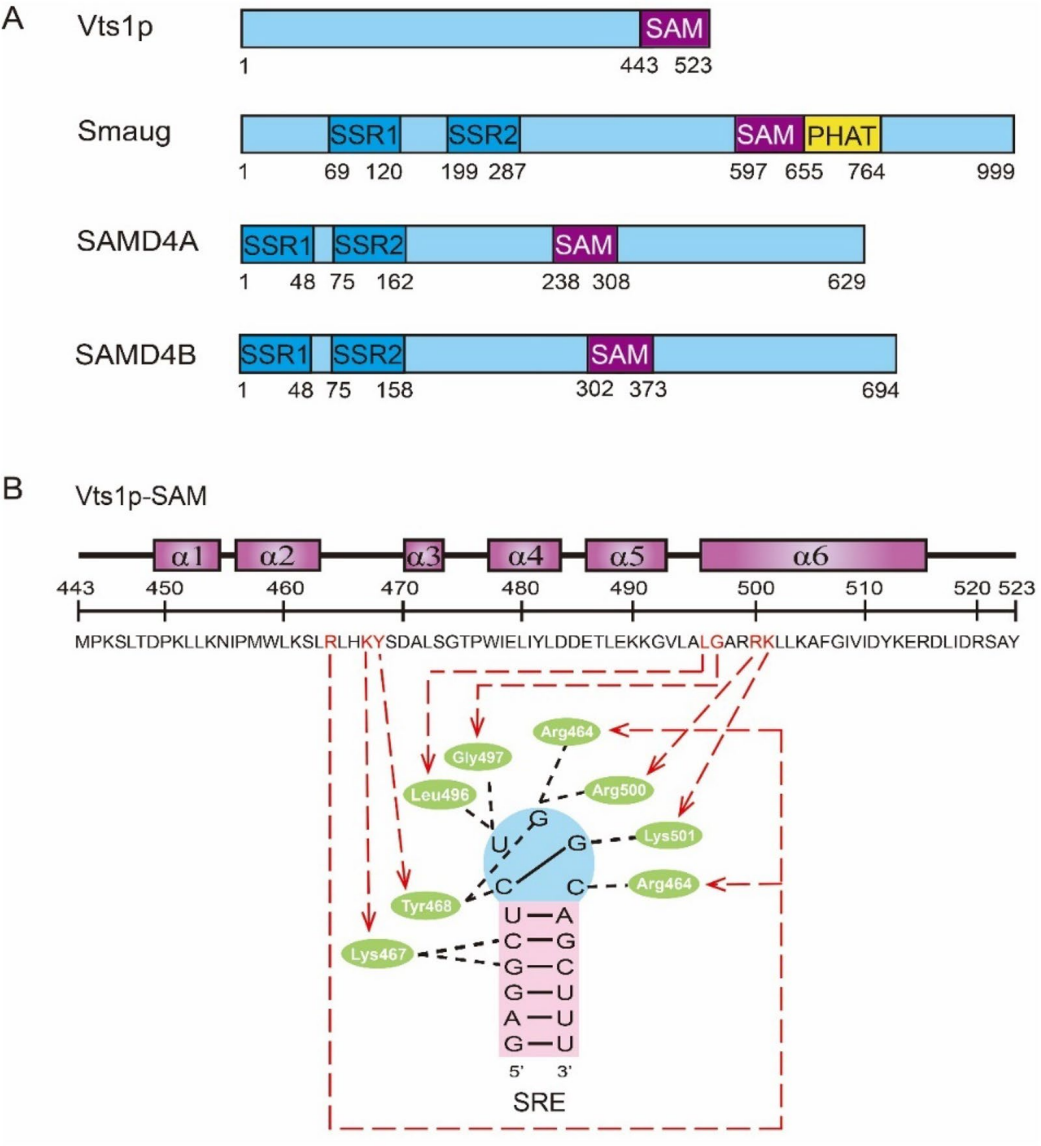
The domain compositions of SAMD4 protein family members and the structural characteristics of yeast Vts1p-SAM domain. **A** The domain compositions of SAMD4 protein members. Yeast Vts1p protein consists of 523 amino acids and one SAM domain. *Drosophila* Smaug protein consisits of 999 amino acids and four domains including SSR1, SSR2, SAM and PHAT domain. Human SAMD4A protein consists of 629 amino acids, while human SAMD4B protein consists of 694 amino acids. Both human SAMD4 proteins contain the common domains including SAM, SSR1, and SSR2 domains. **B** The structual characteristics of yeast Vts1p-SAM domain, which contains six α-helices, seven of these amino acids bind to SRE structure through hydrogen bonds. SAM, sterile alpha motif; SSR, Smaug similarity region. PHAT, pseudo heat analogous topology domain, which can increase the affinity of the SAM domain for SRE.

SAMD4 is a mammalian homolog of *Drosophila* Smaug protein, which has been identified as a novel RNA binding protein and a conserved translational repressor [1]. Mammalian SAMD4 protein family consists of two members SAMD4A/Smaug1 and SAMD4B/Smaug2. SAMD4 proteins from yeast to humans contain a highly conversed SAM domain, which can directly bind mRNA through a stem-loop structure within 25 ~ 40 nucleotides on the 3ʹ untranslated region (UTR) of target mRNA, also known as Smaug recognition elements (SREs). It is found that SREs usually contains the consensus sequence CNGGN or CNGG to form a 5-base or 4-base ring on target mRNAs [3, 17, 18]. In terms of primary structure, *Drosophila* Smaug protein consists of 999 amino acids [2], and yeast Vts1p has 523 amino acids [19]. However, human SAMD4A protein consists of 629 amino acids [20], and human SAMD4B protein consists of 694 amino acids [21]. Except for the common SAM domain, SAMD4 protein family members also have a Smaug similarity region 1 (SSR1) domain composed of 48 amino acids, and Smaug similarity region 2 (SSR2) domain composed of about 83 amino acids at the N-terminal (Fig. 3A). These two conserved domains have high sequence similarity in *Drosophila* Smaug, mouse and human SAMD4 proteins [6]. SSR1 domain functions as a dimerization domain [22], while the function of SSR2 domain remains unknown. It was found that a missense mutation in SSR2 domain of mouse SAMD4 protein leads to a loss-of-function phenotype of myopathy [11]. Either SSR1 or SSR2 domain is deleted, the interaction between *Drosophilia* Smaug and Smoothened (SMO) protein is blocked, suggesting that both domains are essential for the interaction between Smaug and SMO protein [23].

*Ponting* first discovered and defined the SAM domain as a novel motif in yeast sterile and *Drosophila* polyhomeotic proteins, which can mediate protein-protein interaction to regulate the sexual differentiation of yeast and early embryonic development of *Drosophila*, and also participate in cell signal transduction pathways [24]. In addition, Smaug can recruit various proteins through direct protein-protein interaction, including *Drosophila* Cup [25], CUG triplet repeat RNA binding protein 1 (CUGBP1) [26] and Argonaute 1 (Ago1) [27] to target mRNAs for translational repression and/or mRNA degradation. Except for the characterized roles in protein-protein interaction, SAM domain also has the ability to recognize and bind RNA [17]. For example, the SAM domain of yeast Vts1 protein can specifically bind to the RNA hairpin structure in vitro and in vivo [18, 28, 29], while the SAM domain of yeast MAPKKK Ste11 protein plays an important role in transmitting signals to downstream kinases [30]. At present, the crystal structure of the SAM domain of *Drosophila* Smaug and yeast Vts1p has been characterized. The *Drosophila* Smaug-SAM domain consists of one long α-helix (α5), three short α-helices (α1, α3 and α4) and one 3_10_ helix (h2) [17]. The yeast Vts1p-SAM domain consists of six α-helices. Arg464, Lys467, Tyr468, Leu496, Gly497, Arg500 and Lys501 are the main amino acids that bind SRE structure through hydrogen bonding [18, 19, 28, 31] (Fig. 3B).

SAMD4 protein family members play various biological functions through the conserved SAM domain. The SAM domain of *Drosophila* Smaug binds *nanos* mRNA and repress *nanos* mRNA translation, which has been linked to *Drosophila* early embryonic development [6]. Yeast Vts1p and *Drosophila* Smaug are also reported to be involved in the post-transcriptional regulation of gene expression through the common mechanism of interaction with stem-loop structure of target mRNA [3]. Interestingly, the SAM domain of human SAMD4A and SAMD4B proteins can suppress hepatitis B virus (HBV) replication though targeting the conserved SRE stem-loop of HBV RNA and promoting HBV RNA degradation, which suggesting that human SAMD4 proteins have the anti-HBV function [16].

## Biological functions of SAMD4

**Fig. 4:**
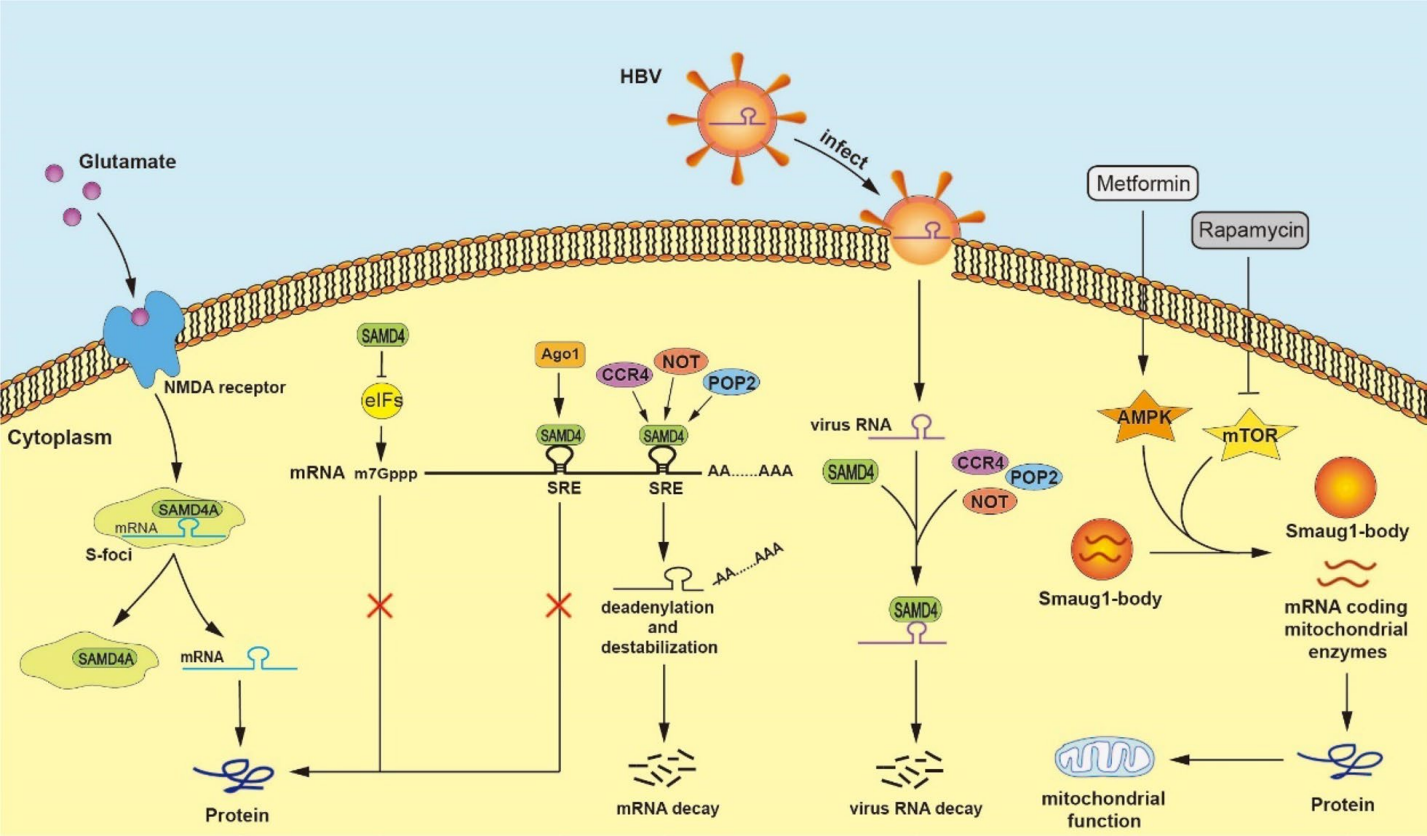
Molecular mechanisms of SAMD4 protein family members of biological functions. From the left to right, the figure describes the molecular mechanisms of SAMD4 family in cytoplasmic foci formation, mRNA decay and translation repression. After glutamate binds to NMDA receptor, mRNA-silencing foci (S foci) are rapidly disassembled and the repressed mRNA is instantaneously released to initiate the translation process. SAMD4 indirectly interacts with eIF4E to repress mRNA translation. SAMD4 recruits Ago1 to target mRNA to inhibit mRNA translation. SAMD4 recruits CCR4/POP2/NOT deadenylase complex to target mRNA and remove poly(A) tail at the 3’-end of mRNA through deadenylation, thus affecting mRNA destabilization and mediating mRNA degradation. The SAM domain of SAMD4 specifically binds to the SRE site in HBV RNA and triggers viral RNA degradation by recruiting CCR4/POP2/NOT deadenylase. AMPK activation by metformin and mTOR inhibition by rapamycin, Smaug1-body releases SDHB and UQCRC1 mRNAs, which are translated into proteins encoding mitochondrial enzymes and participate in the regulation of mitochondria function

There has been a number of related research on SAMD4 family members in the field of RNA binding proteins in recent years. SAMD4 proteins play various biological functions mainly through the conserved SAM domain. *Drosophila* Smaug and mammalian SAMD4 proteins have been identified as a novel post-transcriptional regulator and a new conserved protein translational repressor [3, 6]. In addition, SAMD4 proteins also participate in the formation of cytoplasmic foci [32], and play the antiviral function [16]. The molecular mechanisms by which SAMD4 protein family exerts the specific biological functions are illustrated in Fig. 4.

### Regulation of mRNA stabilization and degradation

Post-transcriptional regulation, mediated by hundreds of RNA binding proteins, is one of the important characteristics of gene expression regulation in the eukaryotes, which includes the splicing and processing of mRNA precursor hnRNA, the process and localization of mRNA from nucleus to cytoplasm, and the stability and degradation of mRNA [33]. Early embryonic development of animals is controlled by RNAs and proteins encoded in the maternal oocyte, and the maturation and activation of oocyte causes changes in the maternal mRNA stability and translation [34]. It is reported that *Drosophila* embryogenesis is programmed by maternal mRNAs [35]. For instance, *Drosophila hsp83* is a homologous gene of mammalian *hsp80*, which can encode a heat shock protein Hsp83. *Drosophila Hsp83* mRNA is initially abundant in adult ovary and embryo, and subsequently passes into the oocyte during 10–11 stage. A large number of *Hsp83* mRNA are accumulated during oogenesis, suggesting that maternal *Hsp83* mRNA plays a critical role in oocytes or early embryos [36]. *Drosophila* Smaug protein can bind target mRNA via the conserved SAM domain that recognizes stem-loop RNA structure termed SREs, and has been defined as a multifunctional post-transcriptional regulator and plays an important role in regulating the stability of these maternal mRNAs in the early *Drosophila* embryo [3, 17]. Smaug can destabilize more than 1000 mRNAs in early embryo, but whether these mRNAs represent direct target mRNAs of Smaug remains unknown. Maternal *Hsp83* mRNA is one of the identified direct target mRNA, but Smaug does not repress *Hsp83* mRNA translation [5]. Studies have shown that *Drosophila* Smaug can degrade *Hsp83* mRNA by binding directly to mRNA through eight SREs in the *Hsp83* open reading frame [37]. Mechanically, *Drosophila* Smaug recruits CCR4/POP2/NOT deadenylase complex to *Hsp83* mRNA, and thus to trigger *Hsp83* transcript destabilization and degradation [12]. It is known that mRNA destabilization mostly depends on mRNA deadenylation in eukaryotes. Shortening of the mRNA poly(A) tails, also called mRNA deadenylation mediated by several deadenylases, is the first and rate-limiting step in the mRNA degradation pathway [38]. In the absence of Smaug, the poly(A) tail of *Hsp83* mRNA are not rapidly shortened, indicating that Smaug is essential for maternal *Hsp83* mRNA deadenylation and further presenting evidence for a role of Smaug in triggering the maternal degradation pathway. Moreover, reduction of CCR4 protein level in the early embryos causes the stabilization of *Hsp83* transcript, further suggesting a role for the deadenylase in mRNA destabilization [12]. Subsequent research demonstrates that mutation of a single amino acid residue on Smaug recognition elements (SREs) can stabilize the endogenous *Hsp83* mRNA, which also indirectly provide evidence for that Smaug directly binds to the *Hsp83* mRNA and plays a key role in regulating mRNA stabilization [37].

Actually, Smaug is essential to degrade a large number of maternal mRNAs in the maternal degradation pathway during early *Drosophila* development [39, 40]. Another study of microarray-based gene expression profiling analysis shows that SREs are strongly enriched during Smaug-dependent degradation, and these maternal mRNAs are directly regulated by Smaug [4]. Therefore, Smaug is a key regulator of maternal mRNA destabilization.

### Regulation of mRNA translation repression

Translational regulation plays an important role in early embryo development and involves RNA binding proteins that interact with elements in the 3’-UTR of specific mRNAs. Smaug, a conversed RNA binding protein, binds to the 3’-UTR sequence of target mRNA to trigger transcript degradation and/or repress mRNA translation. As a novel post-transcriptional regulator, it has been found that Smaug has two identified direct target mRNAs, *Hsp83* and *nanos.* However, Smaug differentially regulates these two maternal target mRNAs through binding to SREs, which further suggesting that Smaug plays an important role in both mRNA destabilization and translation repression. Specifically, Smaug destabilizes and degrades *Hsp83* transcript, but has no detectable effect on *Hsp83* translation [12, 37]. In contrast, Smaug supresses *nanos* mRNA translation through binding two SREs in the 3’-UTR of *nanos* mRNA, but has little effect on *nanos* mRNA stability [1, 2, 6]. Therefore, Smaug is a multifunctional post-transcriptional regulator that can take different mechanisms to regulate maternal mRNA stability and translation.

Except for direct binding to target mRNAs, Smaug recruits various proteins to the target transcripts to repress translation. For example, Smaug can indirectly with eukaryotic initiation factors (eIFs) to regulate mRNA translation. Smaug recruits Cup, an eIF4E-binding protein that blocks the association of eIF4E with eIF4G, and Cup in turn interacts with eIF4E. Cup mediates the indirect interaction between Smaug and eIF4E, and thus to inhibit the initiation of mRNA translation [25]. In the study of Myotonic Dystrophy type 1 (DM1) caused by expansion of untranslated CUG repeats, Smaug is reported as a powerful candidate to modify DM1 pathogenesis using a *Drosophila* DM1 model to screen for genes that suppress CUG-induced toxicity. Increased levels of *smaug* gene in *Drosophila* can prevent muscle dysfunction caused by DM1 mutation. But in human myoblasts, human SAMD4A genetically and physically interacts with CUG triplet repeat RNA binding protein 1 (CUGBP1) and accumulates in cytoplasmic granules. Increasing levels of human SAMD4A can decrease the abnormal nuclear accumulation of CUGBP1 with high steady-state levels in myoblasts from DM1 patients, and also reduce the number of inactive CUGBP1-eIF2α translational complexes. It is suggested that SAMD4A promotes the activity of CUGBP1-containing translation complexes, and SAMD4A can restore the mRNA translation of MORF-related gene on chromosome 15 (MRG15), a target protein of the CUGBP1-eIF2α complex in DM1 myoblasts [26].

In addition, Smaug also directly recruits Argonatue 1 (Ago1) to target mRNA and thereby repress unlocalized *nanos* mRNA translation through binding two SREs in the *nanos* 3’-UTR in a miRNA-independent manner [27]. Ago1 protein is typically recruited to target mRNA by microRNA (miRNA) to form the Ago/miRNA complexes, which can repress translation and or induce mRNA degradation [41]. Smaug can physically interact with Ago1 and Ago1 in turn interacts with *nanos* mRNA. In brief, Smaug protein is necessary for the interaction between Ago1 protein and *nanos* mRNA, but the *nanos* mRNA translational repression does not require the guidance of a targeting miRNA [27, 42].

Recent studies have demonstrated that murine SAMD4A (Smaug1) is associated with the regulation of skeleton development through translational repression of Mig6 protein level. SAMD4A binds to the *Mig6* mRNA and suppress Mig6 protein synthesis. Decreased levels of SAMD4A have increased Mig6 protein expression [15]. However, murine SAMD4B (Smaug2) regulates neurogenesis by inhibiting *nanos1* mRNA translation. Knockdown of SAMD4B increases Nanos1 protein expression and enhances neurogenesis, and the promoting effect on neurogenesis mediated by SAMD4B depletion can be rescued by preventing the increase of Nanos1 protein expression. Thus, murine SAMD4B and Nanos1 proteins function as translational repression switch to regulate neurogenesis [43]. Above these findings strongly clarify the important and regulatory roles of SAMD4 protein family members in the mRNA translation repression.

### Regulation of transcriptional activity

In eukaryotes, transcription initiation is the key point of gene expression regulation. Transcription factors control transcription initiation by binding to a specific DNA sequence to activate or inhibit gene promoter activity [44]. However, in the process of selective expression of genes, the post-transcriptional regulation of genes should not be ignored. It has been found that RNA binding proteins play an essential role as a key regulatory factor in the post-transcriptional regulation, which can participate in each stage from mRNA synthesis to mRNA decay and thereby regulate the activity of mRNA. RNA binding proteins have different affinity and specificity for recognizing and binding target mRNA, and also have a crucial role in all aspects of mRNA metabolism [45, 46].

*Drosophila* Smaug and yeast Vts1p have been defined as multifunctional post-transcriptional regulators partly through a common RNA recognition mechanism [3]. Human SAMD4 protein not only plays an important role in the regulation of post-transcription, but also exhibit the function of regulating the transcriptional activity. One study reported that human SAMD4B is a potential transcriptional repressor and inhibits transcriptional activity [21]. SAMD4B protein is distributed in both the nucleus and cytoplasm, and widely expressed in human adult and embryonic tissues, which is a conserved RNA binding protein during evolution. SAMD4B overexpression inhibits the transcriptional activities of activator protein-1 (AP-1), p21 and p53, and the inhibitory effects can be alleviated by SAMD4B knockdown with small interfering RNA (siRNA). The SAM domain of SAMD4B protein is the main structural region for its role of transcriptional repression, and it is also revealed that SAMD4B acts as a negative transcriptional regulator in the AP-1-p53 signaling pathways [21, 47].

### Formation of mRNA silencing foci (S-foci)

mRNA translational repression is associated with the formation of mRNA silencing foci, such as Processing Bodies (PBs) and Stress Granules (SGs). PBs and SGs are the cytoplasmic RNA granules consisting of repressed mRNAs and proteins, both of which are dynamic and shuttle between the cytoplasm and nucleus. PBs contain the factors related to mRNA degradation and are the storage centers of mRNA, while SGs contain the factors related to translation initiation and are the hubs of signaling events during stress response. The assembly and disassembly of PBs and SGs may play the important role in the regulation of mRNA metabolism [48]. Thus, mRNA silencing foci is a kind of macromolecular aggregates containing silenced mRNAs and their associated proteins, which can release the associated mRNAs and thus to allow their translation according to the cellular needs [49].

Recent studies have first identified a kind of neuron-specific mRNA silencing foci, also named S-foci, which contains a translational repressor of mammalian Smaug1/SAMD4A and is significantly distinct from PBs, SGs and other neuronal RNA granules [50]. Mammalian Smaug1 is expressed in the central nervous system and abundant in the post-synaptic densities, and thus forming post-synaptic mRNA silencing foci in mature hippocampal neurons [51]. S-foci is not the consequence of Smaug1 binding to the repressed mRNAs, and it can respond to synaptic activation. Upon the stimulation of N-methyl-D-aspartic acid (NMDA) receptor, the S-foci can rapidly disassemble and release the repressed mRNAs to initiate their translation and thereby enhance local protein synthesis at the synapse. It is also indicated that mRNA translation is tightly regulated by synaptic stimulation [50, 52]. Conversely, the aggregation of S-foci is also related to whether the contained polyadenylated mRNAs are released from polysomes, suggesting that repressed mRNAs cycle between S-foci and polysomes [32, 52]. Moreover, mammalian Smaug1 is expressed in the process of synaptogenesis, and Smaug1 deletion affects the number and size of synapses, which indicating that Smaug1 regulates local protein translation and affects synapse formation and stability [50, 53]. These findings reveal a role for mammalian Smaug1 in the RNA granule formation and translational regulation of SRE-containing mRNAs in neurons.

### Inhibition of virus replication

In addition to the multifunctional regulatory roles in the post-transcription and translation repression, recent studies have reported that human SAMD4 protein family members also have the function of suppressing hepatitis B virus (HBV) replication [16]. Specifically, *SAMD4A* gene acts as an interferon-stimulated gene (ISG) to inhibit HBV replication and exerts the antiviral effect. Interferon-α (IFN-α) is widely used in the treatment of patients with HBV infection, which is also one of the few drugs approved for clinical treatment of HBV. When patients are infected with hepatitis virus, the expression of ISGs is highly induced in the liver to inhibit the virus replication and thus play the antiviral role [54–56]. It is worth noting that SAMD4B, a homolog of human SAMD4A, can also inhibit HBV replication, but it is not an ISG [16]. Mechanically, the SAM domain of the SAMD4A specifically binds to the conserved SRE-like site in HBV RNA to trigger its degradation and thereby inhibiting HBV replication. In vitro and in vivo experiments showed that both SAMD4A and SAMD4B inhibited HBV replication. In addition, overexpression of SAMD4 in HBV-carrying transgenic mice decreased virus titer, whereas knocking out the *Samd4* gene in hepatocytes increased the HBV replication level in mice [16]. It is suggested that SAMD4A and SAMD4B expression levels are negatively correlated with HBV titers in human hepatitis patients.

It has also been pointed out that the antiviral function of SAMD4 family members is not limited to HBV, maybe SAMD4A has an inhibitory effect on all viruses with mRNA containing the SRE stem loop structure. SAMD4A can recognize and degrade HBV RNA containing the SRE stem loop in the 3′-UTR, which providing the important information for use in IFN therapy of hepatitis B [57, 58].

### Formation of membrane-less organelles (MLOs)

Latest studies have shown that Smaug1 can participate in the formation of cytosolic membrane-less organelles (MLOs), also termed as Smaug1 bodies, and regulate mitochondrial function by responding to AMPK and mTOR signals [59]. Succinate dehydrogenase subunit B (SDHB) and ubiquinol-cytochrome c reductase core protein (UQCRC1) mRNAs that encode the mitochondrial enzymes associate with mammalian Smaug1 MLOs. Smaug1 (SAMD4A) and Smaug2 (SAMD4B) knockdown impairs mitochondrial respiration and disrupts mitochondrial network. Smaug1 MLO dynamics may be affected by mitochondrial activity. Inhibition of mitochondrial complex I by rotenone and inhibition of mTOR by rapamycin, as well as activation of AMPK by metformin induce Smaug1 MLO dissolution and mRNA release. In addition, treatment with Compound C, a known AMPK inhibitor, can block the dissolution of Smaug1 MLO. Smaug1 MLO dissolution leads to the translation of *SDHB* and *UQCRC1* mRNAs invloved in the regulation of mitochondrial function. However, the defective Smaug1 MLO condensation affects the mitochondria funtion. These observations indicate that Smaug1 MLOs respond to AMPK-mTOR balance and affect mitochondrial mRNA release and translation, thus regulating mitochondrial function [59]. It is widely known that the normal funtion of mitochondria is very essential for energy metabolism and cellular functions. Mitochondrial defects also can lead to some diseases, such as cancers, diabets, and several neurological diseases and muscular dystrophies [60, 61]. Therefore, the loss of SAMD4 protein not only leads to the abnormal energy metabolism, but also causes the mitochondrial functional defects.

## SAMD4 and related diseases

In recent years, many studies have shown that SAMD4 protein family is closely related to human diseases. SAMD4 family members can participate in the regulation of the occurrence and development of a variety of diseases, such as myopathy, bone development, neurological diseases and some types of cancers. For instance, it is found that SAMD4 is highly expressed in ovarian cancer and SAMD4B is also highly expressed in colorectal cancer, while SAMD4A is low expressed in breast cancer and SAMD4A is also correlated to the development of oral cancer. In addition, SAMD4 family members are also closely associated with brain aging, obesity, and acute myeloid leukemia. Therefore, it is of great significance to reveal the relationships and regulatory mechanisms between SAMD4 family members and diseases for the prevention and treatment of these diseases.

### SAMD4 and myopathy

There are many types of myopathies, such as congenital myopathy, metabolic myopathy, acquired inflammatory myopathy and toxic myopathy. Myopathy can lead to muscle weakness but does not cause muscle loss of sensation. One of the main reasons of myopathy is that the coupling between excitation and contraction of skeletal muscle is disrupted [62]. One study has shown that in the expansion of untranslated CUG repeat expansion triggered myopathy, Smaug can suppress CUG-induced toxicity and increased levels of Smaug prevent muscle wasting and dysfunction. Meanwhile, increased levels of human SAMD4A in myoblasts from DM1 patients restores the translation activity of CUGBP1, and thus inhibiting CUG-induced myopathy through physically interacting with CUGBP1. Conversely, decreased levels of SAMD4A aggravates the myopathy phenotype induced by CUG and restores muscle function [26]. Another subsequent study has found that a missense mutation of mouse *Samd4* gene results in leanness, myopathy and uncoupled mitochondrial respiration in homozygous mice. These metabolic defects are associated with dysregulated mTORC1 signaling. SAMD4 may combine with mTORC1 signaling through interaction with 14-3-3 proteins and phosphorylation by Akt [11]. These studies reveal the relationships between SAMD4 and myopathy, but the specific molecular mechanism remains to be further studied.

### SAMD4 and bone development

Bone development occurs through a series of synchronous events that cause the formation of the body scaffold. The repair ability of bone and its surrounding microenvironment can restore the tissue and maintain its functional homeostasis [63]. A recent study has shown RNA binding protein mouse SAMD4 is a novel key regulator of bone development and osteoblastogenesis through translational inhibition of Mig6 protein expression. SAMD4 depletion leads to developmental defects in mice such as delayed bone development and reduced osteogenesis. Mechanism research reveals that SAMD4 binds to the *Mig6* mRNA and inhibits the translation of Mig6 protein. Moreover, it is observed that in SAMD4-deficient cells, the expression level of Mig6 protein is increased and depletion of Mig6 rescues the impaired osteogenesis, as well as chondrocyte defects are also observed. These observations suggest that control of protein translation may be an important mechanism for regulating osteoblast formation and bone development [15]. In addition, it was also found that osteoblast differentiation was positively correlated to the phosphorylation level of SAMD4 protein [64]. These findings are of great significance for bone development and the treatment of metabolic bone diseases.

### SAMD4 and neural development

During the development of the mammalian nervous system, neural stem cells first generate neurons and then glial cells, thus allowing the initial establishment of neural circuits [65]. The strength of connections between neurons controls the brain’s ability to store and process information. Synapses are the important structural basis of neuronal connections. Local translation at the synapse is very critical for synaptic plasticity, and deregulation of local translation affect synapse formation and function, thereby causing the neurological disorders [66]. Studies have found that mammalian Smaug1/SAMD4A forms granules in mature hippocampal neurons (termed as S-foci) and participates in the regulation of synaptic plasticity. Knockdown of Smaug1 has an influence on the number and size of synapses. S-foci can control local translation and affect synaptic plasticity [50]. Mouse Smaug1 protein is expressed in the central nervous system and mainly accumulates in the post-synaptic densities (PSD), which play a role in RNA granule formation and translation regulation in neurons [32, 51]. Another study demonstrated that mouse Smaug2 and Nanos1 are critical regulators of murine developmental neurogenesis. Mouse Smaug2 protein/*nanos1* mRNA complex is also present in cytoplasmic granules in the embryonic neural precursors. The results showed that Smaug2 inhibits neurogenesis and Nanos1 promotes neurogenesis. Knockdown of Smaug2 enhances neurogenesis and increases the Nanos1 protein while Nanos1 knockdown inhibits neurogenesis, suggesting that Smaug2 regulates neurogenesis by silencing *nanos1* mRNA. Therefore, Smaug2 and Nanos1 play the critical function of regulating neurogenesis as a bimodal translational repression switch [43]. These findings support that RNA binding protein SAMD4 family is closely associated with mammalian neuronal development.

### SAMD4 and cancer

SAMD4 protein family is not only involved in several physiological processes, but also associated with cancer occurrence and development. Recent studies have indicated that SAMD4A and SAMD4B expressions are significantly changed in some types of cancers and associated with cancer progression (Table 1).

#### SAMD4 and ovarian cancer

Topotecan (TOP) is a chemotherapeutic drug commonly used in the clinical treatment of ovarian cancer. TOP can act as a non-competitive inhibitor that binds to the enzyme-substrate complex to inhibit the replication and transcription of DNA, and eventually leads to the death of ovarian cancer cells [67]. However, ovarian cancer cells easily develop drug resistance to TOP chemotherapy. In topotecan-resistant ovarian cancer cell lines, it is first observed that *SAMD4* gene is overexpressed, and this is also the first report of *SAMD4* gene in drug resistance of ovarian cancer cells [68, 69]. The discovery provides evidence for that *SAMD4* gene is related to the development of resistance to drugs that are used in chemotherapy of ovarian cancer.

#### SAMD4 and colorectal cancer

Colorectal cancer occurrence is mainly caused by the genetic and epigenetic changes of colon epithelial cells, as well as the activation of oncogenes and the inactivation of tumor suppressor genes [70, 71]. The latest research work demonstrates that miR‑451 suppresses the malignant characteristics of colorectal cancer cells through targeting SAMD4B [72]. MicroRNAs (miRNAs) are a class of regulatory RNAs composed of about 22 nucleotides, which can mediate post-transcriptional inhibition by binding to the 3’-UTR of the targeted mRNA, and thereby regulating gene expression [73]. SAMD4B has been identified as a direct target of miR-451. Dual luciferase reporter assay validated that miR-451 could specifically bind to the 3’-UTR of *SAMD4B* mRNA to down-regulate the expression of SAMD4B, thus inhibiting proliferation and promoting apoptosis of colorectal cancer cells. Conversely, overexpression of SAMD4B attenuated miR-451-induced apoptosis and promoted colorectal cancer progression [72]. This study suggests that miR-451/SAMD4B axis may serve as a new therapeutic target for the treatment of patients with colorectal cancer.

#### SAMD4 and breast cancer

Breast cancer is one of the most common cancers in women, and the incidence rate and mortality of breast cancer patients in the world are also rising at present. Therefore, searching for the effective targets is of great significance for the diagnosis and treatment of breast cancer [74]. Tumor angiogenesis is very important to facilitate tumor progression, and controlling the expression of angiogenesis-related factors may help to control the tumor angiogenesis [75]. Recent studies have found that human SAMD4A acts as a novel breast cancer angiogenesis inhibitor [20]. Specifically, the expression of SAMD4A is significantly reduced in human breast cancer tissues, and the low expression is associated with the poor survival of breast cancer patients. Mechanistically, it is found that overexpression of SAMD4A in breast cancer cells downregulates the expression of proangiogenic genes including *CXCL5*, *ENG*, *IL1β* and *ANGPT1*, and destabilizes the proangiogenic mRNAs by the SAM domain of SAMD4A directly binding to the conserved stem-loop structure in the 3’-UTR of these mRNAs, which leads to inhibition of breast tumor angionegesis and progression. On the contrary, knockdown of SAMD4A increases the mRNA stability of these proangiogenic genes and promotes breast tumor angiogenesis and progression [20]. Collectively, these observations suggest that SAMD4A may be a novel breast tumor suppressor and a promising antiangiogenic target for breast cancer therapy.

#### SAMD4 and oral cancer

Oral cancer is one of the most common malignancies in the world, which is also a complex disease affected by the interaction of genetic and environmental factors. Alcohol and smoked tobacco are the two key risk factors leading to oral cancer [76, 77]. The researchers performed the single nucleotide polymorphism (SNP) analysis of rs1957358 in SAMD4A of 500 patients with oral cancer in India, and found that wild-type thymine (T) mutation to cytosine (C) significantly reduced the risk of oral cancer, such as rs1957358 TT mutation to rs1957358 TC, whereas rs1957358 TT reflected the increased risk [78]. This finding identifies SNPs with susceptibility to oral cancer in high risk populations and makes it possible to screen patients susceptible to oral cancer by SNP analysis.

**Table 1:** Expressions, functions and related factors of SAMD4 family in cancer

Cancer type	SAMD4 members	Expression	Functions	Related factors	Refs
Ovarian cancer Colorectal cancer Breast cancer Oral cancer	SAMD4 SAMD4B SAMD4A SAMD4A	↑ ↑ ↓ —	Drug resistance Proliferation, Migration Tumor angiogenesis —	— m*iR-451* *CXCL5, ENG, IL1*β, ANGPT1 —	[68, 69] [72] [20] [78]

“↑” high expression; “↓” low expression; “—” no relevant research.

### SAMD4 and other diseases

In addition to the related diseases mentioned above, SAMD4 family members also play important roles in brain ageing, obesity and acute myeloid leukemia. In the study of brain ageing in mice, it was found that *polygonatum sibiricum* polysaccharide (PSP) could improve the cognitive function during brain ageing and delay brain ageing through regulating the mRNA level of *SAMD4*, which was closely associated with synaptic activity [14]. Studies in a model of diet-induced obesity showed that the expression of the *SAMD4B* and *GATA6* was significantly increased during the differentiation of stromal vascular cells into mature adipocytes, which suggesting that paternal high fat diet is associated with upregulation of *SAMD4B* and *GATA6* genes [79]. Another study on the whole-genome sequencing analysis of patients with acute myeloid leukemia revealed the recurrent mutations in *SAMD4B* gene [80].

In summary, SAMD4 protein family members are closely related to the occurrence and development of a variety of diseases, but the specific molecular mechanisms still need to be further investigeted in the future.

## Molecular mechanisms of action of SAMD4

**Fig. 5:**
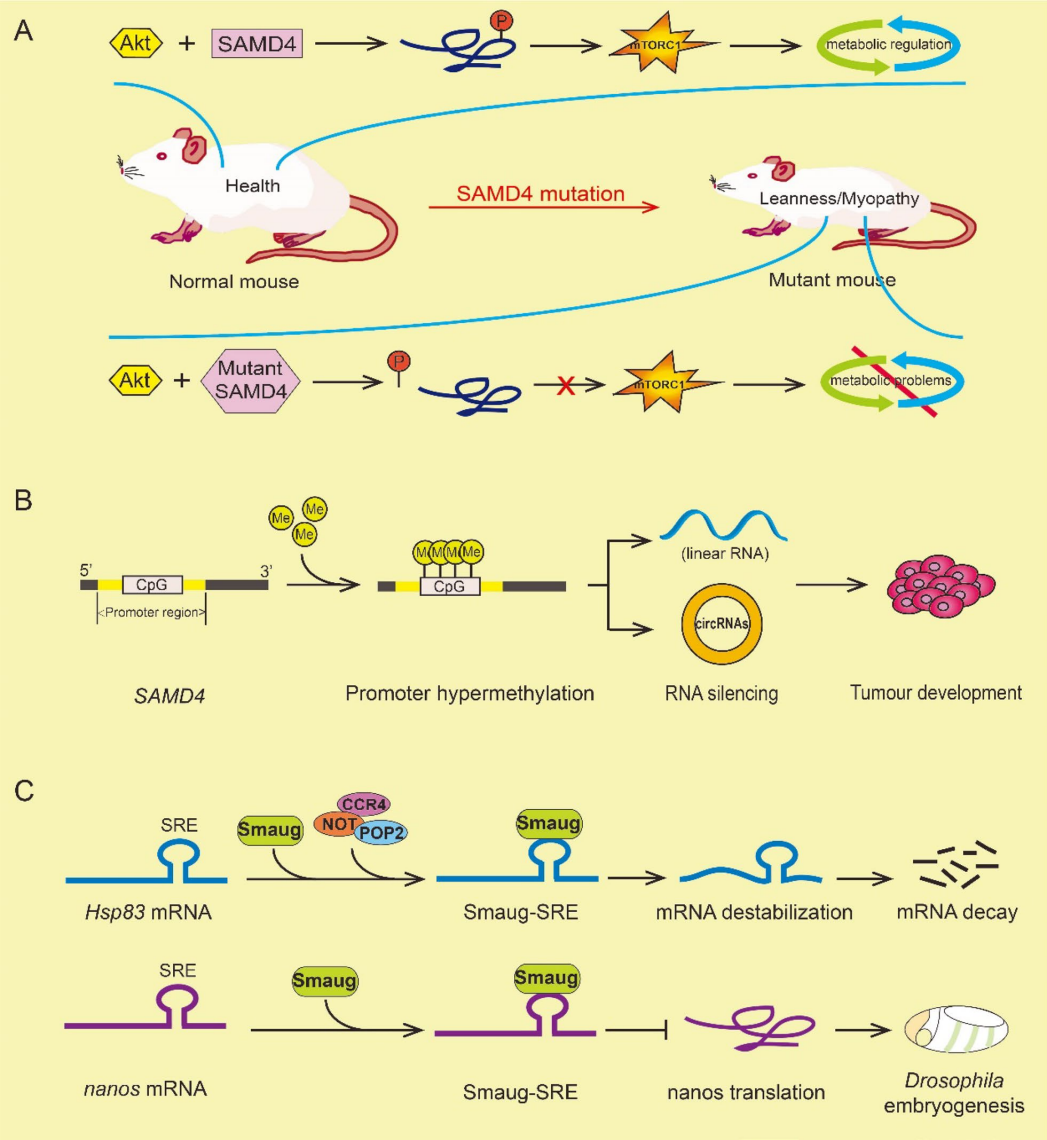
The main factors associated with the SAMD4 family members and their regulatory roles in cellular activities. **A** Interaction between SAMD4 and Akt leads to SAMD4 phosphorylation and affects the downstream mTORC1 signaling and metabolic regulation. **B** Hypermethylation of the CpG island of the SAMD4A gene promoter causes the silencing of circSAMD4A, which regulates tumour development. **C** Smaug recruits CCR4/POP2/NOT deadenylase complex to *Hsp83* mRNA and causes target transcript destabilization and degradation. Smaug binds to *nanos* mRNA to inhibite the nanos protein translation and regulate the *Drosophila* embryogenesis

The biological functions of SAMD4 protein family members require the combined assistance of multiple factors and multiple pathways to regulate the downstream target mRNAs, which can affect the cellular biological functions through regulating transcription and translation (Fig. 5). For instance, SAMD4A can form *circSAMD4A* by back-splicing, which regulates the activities of downstream target mRNAs through sponging various miRNAs, and thus promoting or inhibiting the target protein expression. In addition, SAMD4B is involved in the regulation of p53 signaling pathway. Overexpression of SAMD4B inhibits AP-1, p53 and p21-mediated transcriptional activities, and regulates cell cycle progression and cell apoptosis.

### Action factors associated with SAMD4

Accumulating studies have shown that SAMD4 family members can interact with a variety of factors to play the important biological roles by regulating the downstream signaling pathways or affecting the expression of target genes. SAMD4 directly interacts with Akt, and is phosphorylated by Akt, thereby affecting mTORC1 signaling and playing an important role in metabolic regulation in conjunction with mTORC1 [11]. mTORC1 plays a key role in regulating cell growth and metabolic functions, which can build important molecular connections between nutritional signals and metabolic processes necessary for cell growth. The dysregulation of mTORC1 signaling can lead to a variety of human diseases, such as obesity, diabetes, ageing, neurodegeneration and cancer [81–84]. Therefore, it provides evidence for that SAMD4 is necessary for mTORC1 signaling. After being phosphorylated by Akt, SAMD4 is required for metabolic function through modulating the activities of the mechanistic target of mTORC1 signaling (Fig. 5 A).

Microarray expression profiles of circular RNAs and DNA methylation patterns revealed that promoter CpG island hypermethylation of the hosted circular RNA (circRNA) of *SAMD4A* gene is linked to the transcriptional silencing of the linear mRNA and the hosted circRNA [13]. In general, aberrant methylation in DNA promoter is a very common epigenetic phenomenon in the development of human cancers. Importantly, aberrant methylation of CpG island in DNA promoter is closely related to gene expression regulation [85]. High methylation of DNA promoter can lead to the transcriptional silencing and the inactivation of tumor suppressor genes, which plays a crucial role in tumorigenesis [86]. These findings provide evidence that cancer-specific promoter CpG island hypermethylation silences both the linear and the circular RNAs, which also supporting a role for circRNA hypermethylation-associated epigenetic loss in human cancers (Fig. 5B).

Many studies have demonstrated that SAMD4 family members can interact with various factors to regulate target mRNA degradation or protein translation (Fig. 5 C). For example, *Drosophila* Smaug physically interacts with CCR4/POP2/NOT deadenylase complex in a SRE-independent manner and recruits it to *Hsp83* mRNA to trigger *Hsp83* transcript deadenylation and degradation, but does not repress *Hsp83* translation [12]. In contrast, Smaug represses *nanos* mRNA translation through binding conversed stem-loop structures known as Smaug recognition elements (SREs) in the 3’-UTR of *nanos* mRNA, but does not affect *nanos* mRNA stability [1, 6]. Besides, SAMD4 binds to the SREs in the 3’-UTR of target transcripts for mRNA destabilization and translational repression, such as translational inhibition of Mig6, CXCL5, ENG, IL1β and ANGPT1 expression [15, 87]. Meanwhile, SAMD4 family members can recruit various proteins to the target mRNA for translational repressor and/or transcript degradation. For example, *Drosophila* Smaug can directly interact with eIF4E binding protein CUP and CUP mediates the indirect interaction between Smaug and eIF4E, thereby repressing translation [25]. Smaug can also recruit CUGBP1 to *MRG15* mRNA [26], Ago1 to *nanos* mRNA [27], and G protein-coupled receptor Smoothened (SMO) [23], thereby repressing translation and affecting the cellular physiological activities.

It is worth noting that the transcriptional activity and the expression level of SAMD4 proteins are also regulated by other cellular signalings and biomolecules. For example, *Drosophila* Smaug physically interacts with SMO protein and SMO activation by Hedgehog signaling (HH) causes Smaug phosphorylation. Thus, HH/SMO signaling can both reduce Smaug protein levels and downregulate the repressive activity of *smaug* transcript [23]. In addition, transcriptomic analysis investigates that *Polygonatum sibiricum* polysaccharide (PSP) treatment can regulate the expression level of *SAMD4* mRNA [14]. More interestingly, miR-451 can downregulate the expression of *SAMD4B* both at mRNA and protein levels [72].

### SAMD4A splicing to form circSAMD4A

Circular RNAs (circRNAs) are another new research topic emerging in the field of RNA biology in recent years. CircRNAs are single-stranded, covalently closed endogenous biomolecules and produced by precursor mRNA back-splicing of exons or introns of many genes in eukaryotes, which are generally expressed at low levels and usually show cell type-, tissue-, and developmental stage-specific expression [88, 89]. Actually, circRNAs are a new type of non-coding RNAs (ncRNAs) and are significantly different from traditional linear RNA molecules without 5’-3’ polarities or a poly-adenylated tail, so circRNAs are generally stable and resistant to the digestion of exonuclease [90]. More recently, emerging evidence has indicated that circRNAs can act as microRNA (miRNA) sponges, post-transcriptional regulators of gene expression, and can interact with RNA binding proteins [91, 92]. Moreover, circRNAs contain many miRNA response elements that can allow them specifically bind to multiple miRNAs like sponge, which results in the downregulation of the functional miRNAs and the upregulation of target miRNAs [93, 94]. Importantly, circRNAs have been reported to play crucial roles in the physiological and pathological life processes, and are believed to be the potential diagnostic biomarkers for numerous human diseases, including neurological diseases, cardiovascular diseases, degenerative diseases, lipid disorder diseases and various types of cancers [95–98].

**Fig. 6:**
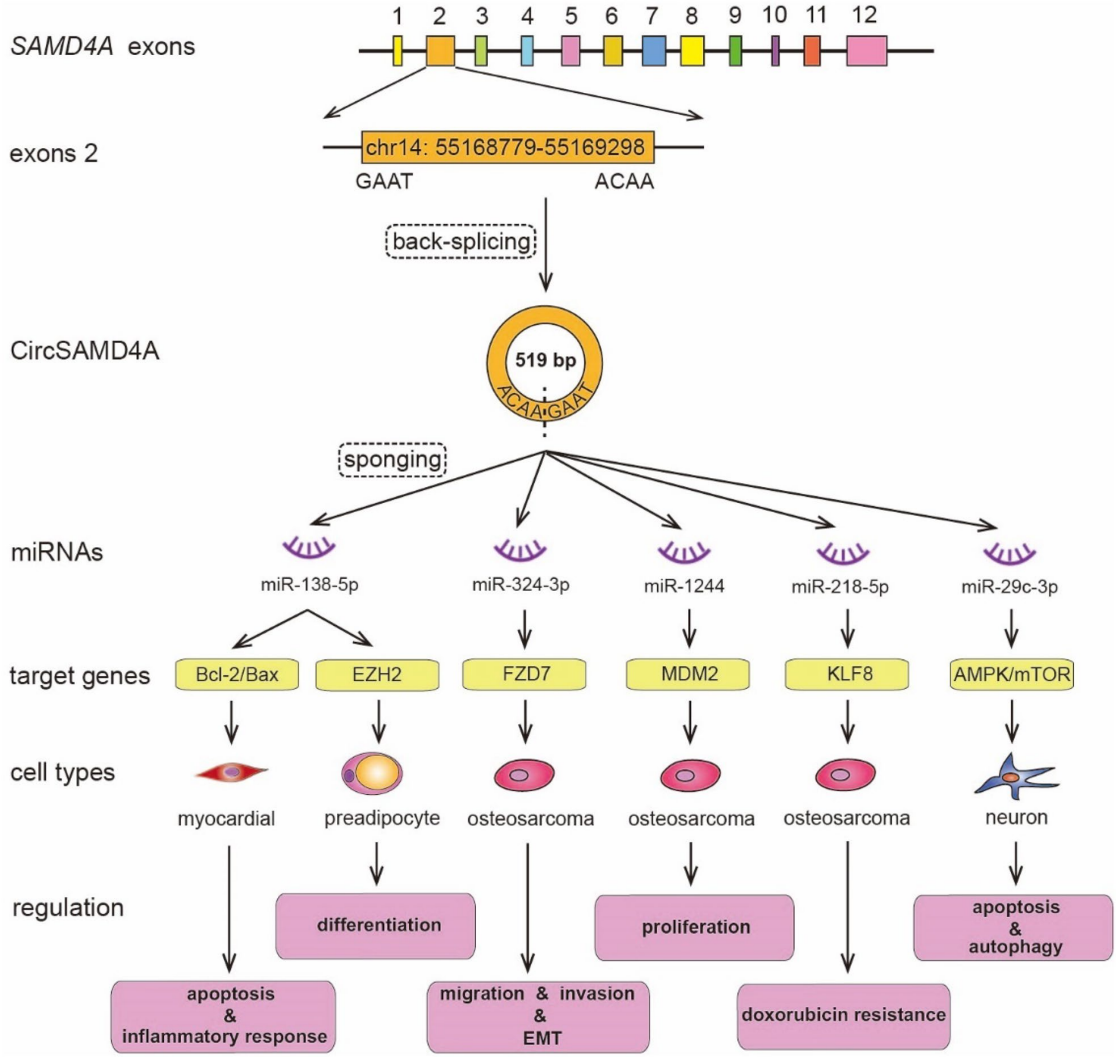
Formation of circSAMD4A and its biological functions in various types of cells. CircSAMD4A is formed by back-splicing of exon 2 of *SAMD4A* gene (chr14: 55,168,779–55,169,298) with a length of 519 bp. CircSAMD4A regulates the biological functions of different types of cells through sponging various miRNAs and regulating the expression of different target genes

RNA binding proteins are necessary for circRNA biogenesis. SAMD4 is a new class of conserved RNA binding protein that acts as a post-transcriptional regulator and translational repressor [2, 3]. Recent studies have discovered that *SAMD4A* gene can form circSAMD4A through back-splicing, and then regulate the activities of *SAMD4A* gene, which is located in chr14:55168779–55,169,298 with the length of 519 bp [99, 100]. Current studies mainly focus on the interaction between circSAMD4A and various miRNAs and its relationship with diseases (Fig. 6). For instance, CircSAMD4A is highly expressed in osteosarcoma (OS) tissues and promotes cell proliferation and enhances cell stemness characteristics by sponging miR-1244 and regulating MDM2 expression in OS, which suggests that circSAMD4A/miR-1244/MDM2 axis may be a promising therapeutic target for OS therapy [99]. Subsequent study showed that circSAMD4A also regulates cell cytotoxicity, migration, invasion, apoptosis and epithelial-to-mesenchymal transition (EMT) through sponging miR-342-3p via the regulation of FDZ7 expression in OS [100]. Another study also demonstrated that circSAMD4A enhances cell doxorubicin (DOX) resistance in OS by regulating the miR-218-5p/KLF8 axis, further suggesting a novel therapeutic target for resisitant OS treatment [101]. In addition, circSAMD4A regulates preadipocyte differentiation through sponging miR-138-5p, and thus upregulating EZH2 expression. These results demonstrate that circSAMD4A controls adipogenesis in obesity via the miR-138-5p/EZH2 axis, and circSAMD4A is associated with obesity and may serve as a potential target for obesity therapy [98, 102]. It is also found that circSAMD4A can sponge miR-138-5p to promote Hypoxia/Reoxygenation (H/R)-induced cardiomyocyte apoptosis and inflammatory response through regulating the expression of Bcl-2/Bax [103]. In Parkinson’s (PD) disease, circSAMD4A is proved to be involved in the regulation of apoptosis and autophagy of dopaminergic neurons through sponging miR‑29c‑3p and modulating the AMPK/mTOR signaling pathway [104]. In addition, recent research also have shown that the expression level of circSAMD4 is significantly increased in the differentiated myoblasts, and depletion of the highly expressed circSAMD4 can delay myogenic progression and affect muscle differentiation, which suggests that circSAMD4 plays an important role in promoting myogenesis as a cytosolic RNA. It is further confirmed that circSAMD4 interacts with myogenic purine-rich binding proteins (PUR) and represses the association of PUR proteins with myosin heavy chain (*Mhc*) promoter sequences, indicating that the interaction between circSAMD4 and PUR proteins promotes myogenesis through derepression of MHC transcription [105]. More interestingly, circSAMD4A is reported to be associated with vascular calcification (VC). Knockdown of circSAMD4A can promote VC, whereas overexpression of circSAMD4A reduces VC. Collectively, these data show that circSAMD4A plays an anti-calcification role through acting as a miRNA sponge [106]. Another study further confirms that circSAMD4A is a novel biomarker for the diagnosis of VC [107].

In addition to acting as miRNA sponges, the latest research has identified the mitochondria-localized circSAMD4 as an important regulator of mitochondrial oxidative stress in cardiomyocyte (CM). Specifically, circSAMD4 overexpression can reduce mitochondrial oxidative stress and oxidative DNA damage. Additionally, circSAMD4 overexpression can induce CM proliferation and prevent CM apoptosis, and thereby improving cardiac function after myocardial infarction (MI) [108]. Reactive oxygen species (ROS) in adult CMs are mainly generated by mitochondria, and mitochondria-localized circRNAs play a cruial role in regulating mitochondria function and dynamics [109, 110]. CircSAMD4 can regulate mitochondrial ROS production and maintain mitochondrial dynamics by inducing the mitochondrial translocation of the Vcp protein and thereby downregulating Vdac1 expression and controlling mitochondrial membrane potential. These findings suggest that circSAMD4 may act as a novel therapeutic target for promoting cardiac regeneration repair and improving cardiac function after MI [108].

### Signaling pathway regulated by SAMD4B

**Fig. 7:**
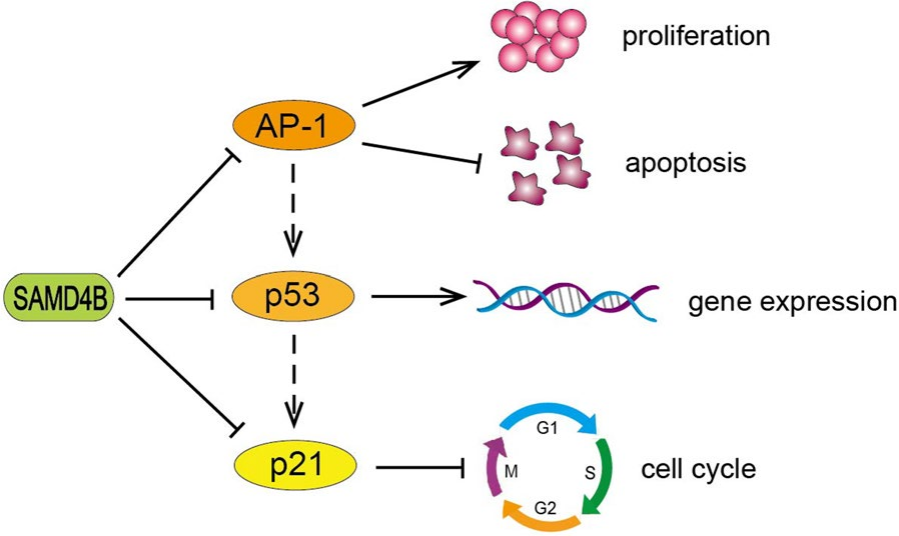
SAMD4B affects the biological functions of cells by regulating p53 signaling pathway. SAMD4B can inhibit the transcriptional activities of AP-1, p53 and p21, thereby promoting cell proliferation and inhibiting cell apoptosis and cell cycle transition, as well as regulating the expression of downstream genes

SAMD4 family members are involved in the regulation of some signaling pathways. At present, the p53 pathway regulated by SAMD4B is clearly studied. The components of p53 pathway are very complex including hundreds of genes and their products, which can respond to many stress signals, regulate the expression of downstream genes, communicate with other signal transduction pathways, and thus affect various cell life processes [47, 111]. Studies have shown that overexpression of *SAMD4B* in mammalian cells inhibits the transcriptional activities of AP-1, p53 and p21, and the effects of transcriptional suppression can be relieved by SAMD4B knockdown with siRNA [21]. p53 is a transcriptional activator that can regulate gene expression in p53 signaling pathway [112]. In the p53 pathway, AP-1 is one of the most important upstream mediators of the p53, which participates in cell proliferation, apoptosis, transformation and differentiation [113]. p21, a downstream component of p53, is a widely recognized tumor suppressor and plays an important role in regulating cell cycle progression as a CDK inhibitor [114, 115]. Therefore, SAMD4B may be a component of the p53 pathway and play a regulatory role in p53 pathway, and thus regulating cell proliferation, cell cycle and apoptosis (Fig. 7).

## Future perspectives

In this review, we briefly summarize the recent progress in the biological functions and molecular mechanisms of SAMD4 family members. SAMD4 proteins are evolutionarily conserved RNA binding proteins and widely expressed in different tissues. To date, SAMD4 family members from different species have attracted a lot of attention from researchers, and gradually become the most important proteins of SAM protein family. The existing studies have revealed that SAMD4 family members play crucial roles in the biological processes through regulating mRNA stability and mRNA degradation, inhibiting transcriptional activity and mRNA translation, participating in the formation of mRNA silencing foci and membrane-less organelles, as well as suppressing virus replication. In addition, SAMD4 family members can interact with multiple factors to regulate various physiological and pathological processes in different types of cells. Strikingly, recent studies have also proved that SAMD4 family members are closely related to the occurrence and development of many human diseases, which may contribute to the diagnosis and clinical treatment of these diseases. These findings greatly expand our knowledge of the biological functions of SAMD4 family members.

Although there are many functional and mechanical studies about SAMD4 protein family members, their molecular regulatory mechanisms have not been fully clarified, and it still faces great challenges. Currently, most of the studies have focused on the *Drosophila* Smaug and mouse SAMD4 animal models, and there are relatively little studies on human SAMD4 proteins, especially the lack of biological functional studies on human SAMD4B protein. Secondly, several studies have reported the relationships between SAMD4 family members and some diseases, but the specific molecular mechanism of regulating diseases needs to be further confirmed. Moreover, current studies have identified some proteins that interacts with SAMD4 family members to participate in the regulation of diseases, whether SAMD4 interacts with other new proteins and plays new functions remain to be further explored. In addition, there are few studies on the signaling pathways regulated by SAMD4 family members, and whether they are also involved in other signaling pathways remains to be investigated. Collectively, there are still many functional and mechanical studies need to be further clarified. SAMD4 family members are favorable potential biomarkers and promising therapeutic targets for some diseases in the future.

## Data Availability

The datasets used and analyzed during the present study are available from the corresponding author on reasonable request.

## Abbreviations

SAMD4: Sterile alpha motif domain containing protein 4
SAM: Sterile alpha motif
RBPs: RNA binding proteins
SSR: Smaug similarity region
SREs: Smaug recognition elements
mRNA: messenger RNA
3ʹ-UTR: 3ʹ-untranslated region
circRNA: circular RNA
miRNA: microRNA
CUGBP1: CUG triplet repeat RNA binding protein 1
Ago1: Argonaute 1
mTORC1: mechanistic target of rapamycin complex 1
SMO: Smoothened
PUR: Purine-rich binding protein
NMDA: N-methyl-d-aspartic acid
MLOs: Membrane-less organelles
OS: Osteosarcoma
CM: Cardiomyocyte

## References

[1] Smibert CA, Wilson JE, Kerr K (1996). Macdonald: smaug protein represses translation of unlocalized nanos mRNA in the Drosophila embryo. Genes Dev.

[2] Dahanukar A, Walker JA, Wharton RP (1999). Smaug, a novel RNA-binding protein that operates a translational switch in Drosophila. Mol Cell.

[3] Aviv T, Lin Z, Lau S, Rendl LM, Sicheri F, Smibert CA (2003). The RNA-binding SAM domain of Smaug defines a new family of post-transcriptional regulators. Nat Struct Biol.

[4] Tadros W, Goldman AL, Babak T, Menzies F, Vardy L, Orr-Weaver T, Hughes TR, Westwood JT, Smibert CA, Lipshitz HD (2007). SMAUG is a major regulator of maternal mRNA destabilization in Drosophila and its translation is activated by the PAN GU kinase. Dev Cell.

[5] Chen L, Dumelie JG, Li X, Cheng MH, Yang Z, Laver JD, Siddiqui NU, Westwood JT, Morris Q, Lipshitz HD, Smibert CA (2014). Global regulation of mRNA translation and stability in the early Drosophila embryo by the Smaug RNA-binding protein. Genome Biol.

[6] Smibert CA, Lie YS, Shillinglaw W, Henzel WJ, Macdonald PM (1999). Smaug, a novel and conserved protein, contributes to repression of nanos mRNA translation in vitro. RNA.

[7] Moore MJ (2005). From birth to death: the complex lives of eukaryotic mRNAs. Science.

[8] Dreyfuss G, Kim VN, Kataoka N (2002). Messenger-RNA-binding proteins and the messages they carry. Nat Rev Mol Cell Biol.

[9] Cammas A, Lewis SM, Vagner S, Holcik M (2008). Post-transcriptional control of gene expression through subcellular relocalization of mRNA binding proteins. Biochem Pharmacol.

[10] Corbett AH (2018). Post-transcriptional regulation of gene expression and human disease. Curr Opin Cell Biol.

[11] Chen Z, Holland W, Shelton JM, Ali A, Zhan X, Won S, Tomisato W, Liu C, Li X, Moresco EM, Beutler B (2014). Mutation of mouse Samd4 causes leanness, myopathy, uncoupled mitochondrial respiration, and dysregulated mTORC1 signaling. Proc Natl Acad Sci U S A.

[12] Semotok JL, Cooperstock RL, Pinder BD, Vari HK, Lipshitz HD, Smibert CA (2005). Smaug recruits the CCR4/POP2/NOT deadenylase complex to trigger maternal transcript localization in the early Drosophila embryo. Curr Biol.

[13] Ferreira HJ, Davalos V, de Moura MC, Soler M, Perez-Salvia M, Bueno-Costa A, Setien F, Moran S, Villanueva A, Esteller M (2018). Circular RNA CpG island hypermethylation-associated silencing in human cancer. Oncotarget.

[14] Zhang Z, Yang B, Huang J, Li W, Yi P, Yi M, Peng W (2021). Identification of the protective effect of Polygonatum sibiricum polysaccharide on d-galactose-induced brain ageing in mice by the systematic characterization of a circular RNA-associated ceRNA network. Pharm Biol.

[15] Niu N, Xiang JF, Yang Q, Wang L, Wei Z, Chen LL, Yang L, Zou W (2017). RNA-binding protein SAMD4 regulates skeleton development through translational inhibition of Mig6 expression. Cell Discov.

[16] Wang Y, Fan X, Song Y, Liu Y, Liu R, Wu J, Li X, Yuan Q, Fu G, Xia N, Han J (2021). SAMD4 family members suppress human hepatitis B virus by directly binding to the Smaug recognition region of viral RNA. Cell Mol Immunol.

[17] Green JB, Gardner CD, Wharton RP, Aggarwal AK (2003). RNA recognition via the SAM domain of Smaug. Mol Cell.

[18] Aviv T, Lin Z, Ben-Ari G, Smibert CA, Sicheri F (2006). Sequence-specific recognition of RNA hairpins by the SAM domain of Vts1p. Nat Struct Mol Biol.

[19] Johnson PE, Donaldson LW (2006). RNA recognition by the Vts1p SAM domain. Nat Struct Mol Biol.

[20] Zhou M, Wang B, Li H, Han J, Li A, Lu W (2021). RNA-binding protein SAMD4A inhibits breast tumor angiogenesis by modulating the balance of angiogenesis program. Cancer Sci.

[21] Luo N, Li G, Li Y, Fan X, Wang Y, Ye X, Mo X, Zhou J, Yuan W, Tan M, Xie H, Ocorr K, Bodmer R, Deng Y, Wu X (2010). SAMD4B, a novel SAM-containing protein, inhibits AP-1-, p53- and p21-mediated transcriptional activity. BMB Rep.

[22] Tang X, Orlicky S, Lin Z, Willems A, Neculai D, Ceccarelli D, Mercurio F, Shilton BH, Sicheri F, Tyers M (2007). Suprafacial orientation of the SCFCdc4 dimer accommodates multiple geometries for substrate ubiquitination. Cell.

[23] Bruzzone L, Argüelles C, Sanial M, Miled S, Alvisi G, Gonçalves-Antunes M, Qasrawi F, Holmgren RA, Smibert CA, Lipshitz HD, Boccaccio GL, Plessis A, Bécam I (2020). Regulation of the RNA-binding protein Smaug by the GPCR Smoothened via the kinase Fused. EMBO Rep.

[24] Ponting CP (1995). SAM: a novel motif in yeast sterile and Drosophila polyhomeotic proteins. Protein Sci.

[25] Nelson MR, Leidal AM, Smibert CA (2004). Drosophila Cup is an eIF4E-binding protein that functions in Smaug-mediated translational repression. Embo j.

[26] de Haro M, Al-Ramahi I, Jones KR, Holth JK, Timchenko LT, Botas J (2013). Smaug/SAMD4A restores translational activity of CUGBP1 and suppresses CUG-induced myopathy. PLoS Genet..

[27] Pinder BD, Smibert CA (2013). microRNA-independent recruitment of Argonaute 1 to nanos mRNA through the Smaug RNA-binding protein. EMBO Rep.

[28] Oberstrass FC, Lee A, Stefl R, Janis M, Chanfreau G, Allain FH (2006). Shape-specific recognition in the structure of the Vts1p SAM domain with RNA. Nat Struct Mol Biol.

[29] Edwards TA, Butterwick JA, Zeng L, Gupta YK, Wang X, Wharton RP, Palmer AG, Aggarwal AK (2006). Solution structure of the Vts1 SAM domain in the presence of RNA. J Mol Biol.

[30] Bhunia A, Ilyas H, Bhattacharjya S (2020). Salt Dependence Conformational Stability of the Dimeric SAM domain of MAPKKK Ste11 from budding yeast: a native-state H/D exchange NMR study. Biochemistry.

[31] Ravindranathan S, Oberstrass FC, Allain FH (2010). Increase in backbone mobility of the VTS1p-SAM domain on binding to SRE-RNA. J Mol Biol.

[32] Baez MV, Boccaccio GL (2005). Mammalian Smaug is a translational repressor that forms cytoplasmic foci similar to stress granules. J Biol Chem.

[33] Gerstberger S, Hafner M, Tuschl T (2014). A census of human RNA-binding proteins. Nat Rev Genet.

[34] Tadros W, Lipshitz HD (2005). Setting the stage for development: mRNA translation and stability during oocyte maturation and egg activation in Drosophila. Dev Dyn.

[35] Bashirullah A, Halsell SR, Cooperstock RL, Kloc M, Karaiskakis A, Fisher WW, Fu W, Hamilton JK, Etkin LD, Lipshitz HD (1999). Joint action of two RNA degradation pathways controls the timing of maternal transcript elimination at the midblastula transition in Drosophila melanogaster. Embo J.

[36] Zimmerman JL, Petri W, Meselson M (1983). Accumulation of a specific subset of D. melanogaster heat shock mRNAs in normal development without heat shock. Cell.

[37] Semotok JL, Luo H, Cooperstock RL, Karaiskakis A, Vari HK, Smibert CA (2008). Lipshitz: Drosophila maternal Hsp83 mRNA destabilization is directed by multiple SMAUG recognition elements in the open reading frame. Mol Cell Biol.

[38] Yan YB (2014). Deadenylation: enzymes, regulation, and functional implications. Wiley Interdiscip Rev RNA.

[39] Thomsen S, Anders S, Janga SC, Huber W, Alonso CR (2010). Genome-wide analysis of mRNA decay patterns during early Drosophila development. Genome Biol.

[40] Götze M, Wahle E (2014). Smaug destroys a huge treasure. Genome Biol.

[41] Hammell CM (2008). The microRNA-argonaute complex: a platform for mRNA modulation. RNA Biol.

[42] Pinder BD, Smibert CA (2013). Smaug: an unexpected journey into the mechanisms of post-transcriptional regulation. Fly (Austin).

[43] Amadei G, Zander MA, Yang G, Dumelie JG, Vessey JP, Lipshitz HD, Smibert CA, Kaplan DR, Miller FD (2015). A Smaug2-Based translational repression Complex determines the balance between precursor maintenance versus differentiation during mammalian neurogenesis. J Neurosci.

[44] Gaston K, Jayaraman PS (2003). Transcriptional repression in eukaryotes: repressors and repression mechanisms. Cell Mol Life Sci.

[45] Müller-McNicoll M, Rossbach O, Hui J, Medenbach J (2019). Auto-regulatory feedback by RNA-binding proteins. J Mol Cell Biol.

[46] Kishore S, Luber S, Zavolan M (2010). Deciphering the role of RNA-binding proteins in the post-transcriptional control of gene expression. Brief Funct Genomics.

[47] Levine AJ, Hu W, Feng Z (2006). The P53 pathway: what questions remain to be explored?. Cell Death Differ.

[48] Riggs CL, Kedersha N, Ivanov P, Anderson P (2020). Mammalian stress granules and P bodies at a glance. J Cell Sci..

[49] Thomas MG, Loschi M, Desbats MA, Boccaccio GL (2011). RNA granules: the good, the bad and the ugly. Cell Signal.

[50] Baez MV, Luchelli L, Maschi D, Habif M, Pascual M, Thomas MG, Boccaccio GL (2011). Smaug1 mRNA-silencing foci respond to NMDA and modulate synapse formation. J Cell Biol.

[51] Fernández-Alvarez AJ, Pascual ML, Boccaccio GL, Thomas MG (2016). Smaug variants in neural and non-neuronal cells. Commun Integr Biol..

[52] Pascual ML, Luchelli L, Habif M, Boccaccio GL (2012). Synaptic activity regulated mRNA-silencing foci for the fine tuning of local protein synthesis at the synapse. Commun Integr Biol.

[53] Thomas MG, Boccaccio GL (2016). Novel mRNA-silencing bodies at the synapse: a never-ending story. Commun Integr Biol..

[54] Yue X, Wang H, Zhao F, Liu S, Wu J, Ren W, Zhu Y (2012). Hepatitis B virus-induced calreticulin protein is involved in IFN resistance. J Immunol.

[55] Wieland SF, Asabe S, Engle RE, Purcell RH, Chisari FV (2014). Limited hepatitis B virus replication space in the chronically hepatitis C virus-infected liver. J Virol.

[56] Alfaiate D, Lucifora J, Abeywickrama-Samarakoon N, Michelet M, Testoni B, Cortay JC, Sureau C, Zoulim F, Dény P, Durantel D (2016). HDV RNA replication is associated with HBV repression and interferon-stimulated genes induction in super-infected hepatocytes. Antiviral Res.

[57] Schoggins JW, Wilson SJ, Panis M, Murphy MY, Jones CT, Bieniasz P, Rice CM (2011). A diverse range of gene products are effectors of the type I interferon antiviral response. Nature.

[58] Hubel P, Urban C, Bergant V, Schneider WM, Knauer B, Stukalov A, Scaturro P, Mann A, Brunotte L, Hoffmann HH, Schoggins JW, Schwemmle M, Mann M, Rice CM, Pichlmair A (2019). A protein-interaction network of interferon-stimulated genes extends the innate immune system landscape. Nat Immunol.

[59] Fernández-Alvarez AJ, Gabriela Thomas M, Pascual ML, Habif M, Pimentel J, Corbat AA, Pessoa JP, La Spina PE, Boscaglia L, Plessis A, Carmo-Fonseca M, Grecco HE, Casado M, Boccaccio GL (2022). Smaug1 membrane-less organelles respond to AMPK and mTOR and affect mitochondrial function. J Cell Sci.

[60] Schapira AH (2012). Mitochondrial diseases. Lancet.

[61] Srinivasan S, Guha M, Kashina A, Avadhani NG (2017). Mitochondrial dysfunction and mitochondrial dynamics-the cancer connection. Biochim Biophys Acta Bioenerg.

[62] Chiodo A (2013). Acquired myopathy/dystrophies. Pm R.

[63] Salhotra A, Shah HN, Levi B, Longaker MT (2020). Mechanisms of bone development and repair. Nat Rev Mol Cell Biol.

[64] Cao J, Man Y, Li L (2013). Electrical stimuli improve osteogenic differentiation mediated by aniline pentamer and PLGA nanocomposites. Biomed Rep.

[65] Miller FD, Gauthier AS (2007). Timing is everything: making neurons versus glia in the developing cortex. Neuron.

[66] Jackman SL, Regehr WG (2017). The mechanisms and functions of synaptic facilitation. Neuron.

[67] Staker BL, Hjerrild K, Feese MD, Behnke CA, Burgin AB, Stewart L (2002). The mechanism of topoisomerase I poisoning by a camptothecin analog. Proc Natl Acad Sci U S A.

[68] Klejewski A, Świerczewska M, Zaorska K, Brązert M, Nowicki M, Zabel M, Januchowski R (2017). New and Old genes Associated with Topotecan Resistance Development in Ovarian Cancer Cell Lines. Anticancer Res.

[69] Januchowski R, Sterzyńska K, Zawierucha P, Ruciński M, Świerczewska M, Partyka M, Bednarek-Rajewska K, Brązert M, Nowicki M, Zabel M, Klejewski A (2017). Microarray-based detection and expression analysis of new genes associated with drug resistance in ovarian cancer cell lines. Oncotarget.

[70] Das V, Kalita J, Pal M (2017). Predictive and prognostic biomarkers in colorectal cancer: a systematic review of recent advances and challenges. Biomed Pharmacother.

[71] Wang FW, Cao CH, Han K, Zhao YX, Cai MY, Xiang ZC, Zhang JX, Chen JW, Zhong LP, Huang Y, Zhou SF, Jin XH, Guan XY, Xu RH, Xie D (2019). APC-activated long noncoding RNA inhibits colorectal carcinoma pathogenesis through reduction of exosome production. J Clin Invest.

[72] Wu C, Liu X, Li B, Sun G, Peng C, Xiang D (2021). miR–451 suppresses the malignant characteristics of colorectal cancer via targeting SAMD4B. Mol Med Rep..

[73] Chen CZ, Li L, Lodish HF, Bartel DP (2004). MicroRNAs modulate hematopoietic lineage differentiation. Science.

[74] Ullah MF (2019). Breast Cancer: current perspectives on the Disease Status. Adv Exp Med Biol.

[75] Jiang X, Wang J, Deng X, Xiong F, Zhang S, Gong Z, Li X, Cao K, Deng H, He Y, Liao Q, Xiang B, Zhou M, Guo C, Zeng Z, Li G, Li X, Xiong W (2020). The role of microenvironment in tumor angiogenesis. J Exp Clin Cancer Res.

[76] Montero PH, Patel SG (2015). Cancer of the oral cavity. Surg Oncol Clin N Am.

[77] Nagao T, Warnakulasuriya S (2020). Screening for oral cancer: future prospects, research and policy development for Asia. Oral Oncol.

[78] D’Souza W, Pradhan S, Saranath D (2017). Multiple single nucleotide polymorphism analysis and association of specific genotypes in FHIT, SAMD4A, and ANKRD17 in indian patients with oral cancer. Head Neck.

[79] Bernhardt L, Dittrich M, El-Merahbi R, Saliba AE, Müller T, Sumara G, Vogel J, Nichols-Burns S, Mitchell M, Haaf T, El Hajj N (2021). A genome-wide transcriptomic analysis of embryos fathered by obese males in a murine model of diet-induced obesity. Sci Rep.

[80] Kayser S, Hills RK, Langova R, Kramer M, Guijarro F, Sustkova Z, Estey EH, Shaw CM, Ráčil Z, Mayer J, Zak P, Baer MR, Brunner AM, Szotkowski T, Cetkovsky P, Grimwade D, Walter RB, Burnett AK, Ho AD, Ehninger G, Müller-Tidow C, Platzbecker U, Thiede C, Röllig C, Schulz A, Warsow G, Brors B, Esteve J, Russell NH, Schlenk RF, Levis MJ (2021). Characteristics and outcome of patients with acute myeloid leukaemia and t(8;16)(p11;p13): results from an International Collaborative Study. Br J Haematol.

[81] Laplante M, Sabatini DM (2012). mTOR signaling in growth control and disease. Cell.

[82] Ben-Sahra I, Manning BD (2017). mTORC1 signaling and the metabolic control of cell growth. Curr Opin Cell Biol.

[83] González A, Hall MN (2017). Nutrient sensing and TOR signaling in yeast and mammals. Embo j.

[84] Liu GY, Sabatini DM (2020). mTOR at the nexus of nutrition, growth, ageing and disease. Nat Rev Mol Cell Biol.

[85] Morgan AE, Davies TJ, Mc Auley MT (2018). The role of DNA methylation in ageing and cancer. Proc Nutr Soc.

[86] Kulis M, Esteller M (2010). DNA methylation and cancer. Adv Genet.

[87] Zhou M, Wang B, Li H, Han J, Li A, Lu W (2021). RNA-binding protein SAMD4A inhibits breast tumor angiogenesis by modulating the balance of angiogenesis program. Cancer Sci.

[88] Kristensen LS, Andersen MS, Stagsted LVW, Ebbesen KK, Hansen TB, Kjems J (2019). The biogenesis, biology and characterization of circular RNAs. Nat Rev Genet.

[89] Li X, Yang L, Chen LL (2018). The Biogenesis, Functions, and Challenges of Circular RNAs. Mol Cell.

[90] Qu S, Yang X, Li X, Wang J, Gao Y, Shang R, Sun W, Dou K, Li H (2015). Circular RNA: a new star of noncoding RNAs. Cancer Lett.

[91] Lasda E, Parker R (2014). Circular RNAs: diversity of form and function. RNA.

[92] Zhou WY, Cai ZR, Liu J, Wang DS, Ju HQ, Xu RH (2020). Circular RNA: metabolism, functions and interactions with proteins. Mol Cancer.

[93] Memczak S, Jens M, Elefsinioti A, Torti F, Krueger J, Rybak A, Maier L, Mackowiak SD, Gregersen LH, Munschauer M, Loewer A, Ziebold U, Landthaler M, Kocks C (2013). le Noble and N. Rajewsky: circular RNAs are a large class of animal RNAs with regulatory potency. Nature.

[94] Tay Y, Rinn J, Pandolfi PP (2014). The multilayered complexity of ceRNA crosstalk and competition. Nature.

[95] Qu S, Zhong Y, Shang R, Zhang X, Song W, Kjems J, Li H (2017). The emerging landscape of circular RNA in life processes. RNA Biol.

[96] Zhang Z, Yang T, Xiao J (2018). Circular RNAs: promising biomarkers for Human Diseases. EBioMedicine.

[97] Zhong Y, Du Y, Yang X, Mo Y, Fan C, Xiong F, Ren D, Ye X, Li C, Wang Y, Wei F, Guo C, Wu X, Li X, Li Y, Li G, Zeng Z, Xiong W (2018). Circular RNAs function as ceRNAs to regulate and control human cancer progression. Mol Cancer.

[98] Yu G, Yang Z, Peng T, Lv Y (2021). Circular RNAs: rising stars in lipid metabolism and lipid disorders. J Cell Physiol.

[99] Yanbin Z, Jing Z (2019). CircSAMD4A accelerates cell proliferation of osteosarcoma by sponging miR-1244 and regulating MDM2 mRNA expression. Biochem Biophys Res Commun.

[100] Xie C, Chen B, Wu B, Guo J, Shi Y, Cao Y (2020). CircSAMD4A regulates cell progression and epithelial–mesenchymal transition by sponging miR–342–3p via the regulation of FZD7 expression in osteosarcoma. Int J Mol Med.

[101] Wei W, Ji L, Duan W, Zhu J (2020). CircSAMD4A contributes to cell doxorubicin resistance in osteosarcoma by regulating the miR-218-5p/KLF8 axis. Open Life Sci.

[102] Liu Y, Liu H, Li Y, Mao R, Yang H, Zhang Y, Zhang Y, Guo P, Zhan D, Zhang T (2020). Circular RNA SAMD4A controls adipogenesis in obesity through the miR-138-5p/EZH2 axis. Theranostics.

[103] Hu X, Ma R, Cao J, Du X, Cai X, Fan Y (2020). CircSAMD4A aggravates H/R-induced cardiomyocyte apoptosis and inflammatory response by sponging miR-138-5p. J Cell Mol Med.

[104] Wang W, Lv R, Zhang J, Liu Y (2021). circSAMD4A participates in the apoptosis and autophagy of dopaminergic neurons via the miR–29c–3p–mediated AMPK/mTOR pathway in Parkinson’s disease. Mol Med Rep.

[105] Pandey PR, Yang JH, Tsitsipatis D, Panda AC, Noh JH, Kim KM, Munk R, Nicholson T, Hanniford D, Argibay D, Yang X, Martindale JL, Chang MW, Jones SW, Hernando E, Sen P, De S, Abdelmohsen K, Gorospe M (2020). circSamd4 represses myogenic transcriptional activity of PUR proteins. Nucleic Acids Res.

[106] Ryu J, Kwon DH, Choe N, Shin S, Jeong G, Lim YH, Kim J, Park WJ, Kook H, Kim YK (2020). Characterization of circular RNAs in vascular smooth muscle cells with vascular calcification. Mol Ther Nucleic Acids.

[107] Zhou Y, Liu Y, Xuan S, Jin T, Chen K, Wu Z, Su W, Chen L, Zong G (2022). CircSamd4: a novel biomarker for predicting vascular calcification. J Clin Lab Anal..

[108] Zheng H, Huang S, Wei G, Sun Y, Li C, Si X, Chen Y, Tang Z, Li X, Chen Y, Liao W, Liao Y, Bin J (2022). CircRNA Samd4 induces cardiac repair after myocardial infarction by blocking mitochondria-derived ROS output. Mol Ther.

[109] Zhang D, Li Y, Heims-Waldron D, Bezzerides V, Guatimosim S, Guo Y, Gu F, Zhou P, Lin Z, Ma Q, Liu J, Wang DZ (2018). Pu: mitochondrial cardiomyopathy caused by elevated reactive oxygen species and impaired cardiomyocyte proliferation. Circ Res.

[110] van Heesch S, Witte F, Schneider-Lunitz V, Schulz JF, Adami E, Faber AB, Kirchner M, Maatz H, Blachut S, Sandmann CL, Kanda M, Worth CL, Schafer S, Calviello L, Merriott R, Patone G, Hummel O, Wyler E, Obermayer B, Mücke MB, Lindberg EL, Trnka F, Memczak S, Schilling M, Felkin LE, Barton PJR, Quaife NM, Vanezis K, Diecke S, Mukai M, Mah N, Oh SJ, Kurtz A, Schramm C, Schwinge D, Sebode M, Harakalova M, Asselbergs FW, Vink A, de Weger RA, Viswanathan S, Widjaja AA, Gärtner-Rommel A, Milting H, Dos Remedios C, Knosalla C, Mertins P, Landthaler M, Vingron M, Linke WA, Seidman JG, Seidman CE, Rajewsky N, Ohler U (2019). S. A. Cook and N. Hubner: the Translational Landscape of the Human Heart. Cell.

[111] Harris SL, Levine AJ (2005). The p53 pathway: positive and negative feedback loops. Oncogene.

[112] Giaccia AJ, Kastan MB (1998). The complexity of p53 modulation: emerging patterns from divergent signals. Genes Dev.

[113] Shaulian E, Karin M (2001). AP-1 in cell proliferation and survival. Oncogene.

[114] Gartel AL, Serfas MS (1996). Tyner: p21–negative regulator of the cell cycle. Proc Soc Exp Biol Med.

[115] Sherr CJ, Roberts JM (1999). CDK inhibitors: positive and negative regulators of G1-phase progression. Genes Dev.

